# The genome sequence of the Dwarf Willow, *Salix herbacea* L. (Malpighiales: Salicaceae)

**DOI:** 10.12688/wellcomeopenres.28051.1

**Published:** 2026-10-05

**Authors:** Markus Ruhsam, Peter M. Hollingsworth, Royal Botanic Garden Edinburgh Genome Acquisition Lab, Plant Genome Sizing Collective, Darwin Tree of Life Barcoding collective, Wellcome Sanger Institute Tree of Life Management, Samples and Laboratory team, Wellcome Sanger Institute Scientific Operations: Sequencing Operations, Wellcome Sanger Institute Tree of Life Core Informatics team, Tree of Life Core Informatics collective, Darwin Tree of Life Consortium

**Affiliations:** 1 Royal Botanic Garden Edinburgh, Edinburgh, Scotland, UK

**Keywords:** Salix herbacea, Dwarf Willow, genome sequence, chromosomal, Malpighiales

## Abstract

We present a genome assembly of *Salix herbacea* (Dwarf Willow; Streptophyta; Magnoliopsida; Malpighiales; Salicaceae). The primary assembly has a total length of 421.43 megabases. Most of the primary assembly (92.79%) is scaffolded into 19 chromosomal pseudomolecules. The mitochondrial genome assembly has a length of 692.62 kilobases and the plastid genome assembly has a length of 155.63 kilobases. This assembly was generated as part of the Darwin Tree of Life project, which produces reference genomes for eukaryotic species found in Britain and Ireland.

## Species taxonomy

Eukaryota; Viridiplantae; Streptophyta; Streptophytina; Embryophyta; Tracheophyta; Euphyllophyta; Spermatophyta; Magnoliopsida; Mesangiospermae; eudicotyledons; Gunneridae; Pentapetalae; rosids; fabids; Malpighiales; Salicaceae; Saliceae; *Salix*; *Salix herbacea* L. (NCBI:txid77064)

## Background

*Salix herbacea* is a prostrate, deciduous dwarf shrub with an Arctic–alpine distribution native to Europe, the westernmost parts of Asia, Greenland and North America ( Beerling, 1998). In Britain and Ireland it is widespread only in Scotland, with localised occurrences in England, Wales and Ireland ( Beerling, 1998). It is a species of predominantly montane habitats, occurring in open vegetation on windswept summits and ridges, late-lying snow-beds, corries, screes, erosion surfaces and rock ledges, generally on acidic, nutrient-poor substrates ( Beerling, 1998).

Like most willows, *Salix herbacea* is dioecious, with male and female flowers borne on separate individuals. Its small, few-flowered catkins are produced at the ends of short leafy shoots and generally emerge alongside the developing leaves. Willows may be pollinated by both insects and wind, although the relative importance of these mechanisms in *S. herbacea* remains uncertain ( Beerling, 1998). Because the species is dioecious, the spatial distribution and relative abundance of male and female plants can strongly influence reproductive success. Small or isolated populations may experience reduced pollen transfer, fruit set and seed production when flowering individuals of the opposite sex are scarce or widely separated. Together with limited seedling establishment, these reproductive constraints can increase reliance on vegetative growth and contribute to the dominance of a small number of clones in isolated populations ( Carbognani *et al.*, 2019).

Reproduction occurs both sexually, through seeds, and vegetatively through the extension and fragmentation of the subterranean stem system. Most of the woody branching system of *Salix herbacea* lies at or below the soil surface, with only short leafy shoots projecting above the surrounding vegetation ( Beerling, 1998). The extensive horizontal stems branch and root repeatedly, producing prostrate mats that may extend over substantial areas. Sections of this system can become physically separated through decay or disturbance, allowing the resulting ramets to persist independently while remaining identical components of the same genet ( Beerling, 1998; Centenaro *et al.* , 2023).

However, population-genetic studies show that the relative contributions of sexual and vegetative reproduction vary among populations. In the Scottish population examined by Stamati *et al.*. (2007), most sampled stems had distinct multilocus genotypes, indicating that establishment from seed had contributed substantially to population structure despite the species’ capacity for clonal spread. Other alpine populations contained fewer, spatially extensive genets, demonstrating a greater contribution from vegetative reproduction ( Reisch *et al.*, 2007). Sexual recruitment may be particularly restricted in small populations near the southern limit of the species’ range, where low population density and spatial separation of the sexes can limit reproductive success and increase reliance on clonal persistence ( Carbognani *et al.*, 2019). Once established, individual clones can be exceptionally long-lived. Genetic and growth-ring analyses of *S. herbacea* in the northern Apennines indicate minimum ages exceeding 2,000 years for some genets, placing them among the oldest directly dated clonal woody plants ( Centenaro *et al.* , 2023).

Plant form and phenology vary with snow duration and exposure. In a reciprocal-transplant experiment, plants in early-melting ridge habitats developed leaves earlier but produced smaller leaves than those in later-melting snow-beds, demonstrating substantial phenotypic plasticity. No consistent home-site advantage or significant response of sexual or clonal reproduction to altered snowmelt timing was detected ( Sedlacek *et al.* , 2015).

*Salix herbacea* forms ectomycorrhizal associations with a diverse community of fungi. Molecular analyses of roots from alpine tundra have identified numerous ectomycorrhizal taxa and found substantial overlap between the fungal communities associated with *S. herbacea* and *S. polaris* across contrasting alpine habitats ( Ryberg *et al.*, 2011). These associations are likely to be particularly important for nutrient acquisition in cold, acidic soils where decomposition and nutrient mineralisation are slow.

*Salix herbacea* is diploid, with a chromosome number of 2 *n* = 38. This chromosome number has been consistently reported in geographically separated European and North American populations ( CCDB, 2026; Izmailow, 1980; Wilkinson, 1944; Rice *et al.*, 2015, and references therein).

We sequenced the genome of *Salix herbacea* as part of the Darwin Tree of Life Project, which aims to generate high-quality reference genomes for all named eukaryotic species in Britain and Ireland to support research, conservation, and the sustainable use of biodiversity. The sequenced specimen was collected from Fraoch-bheinn, Scotland, UK (Figure 1).

**Figure 1.**
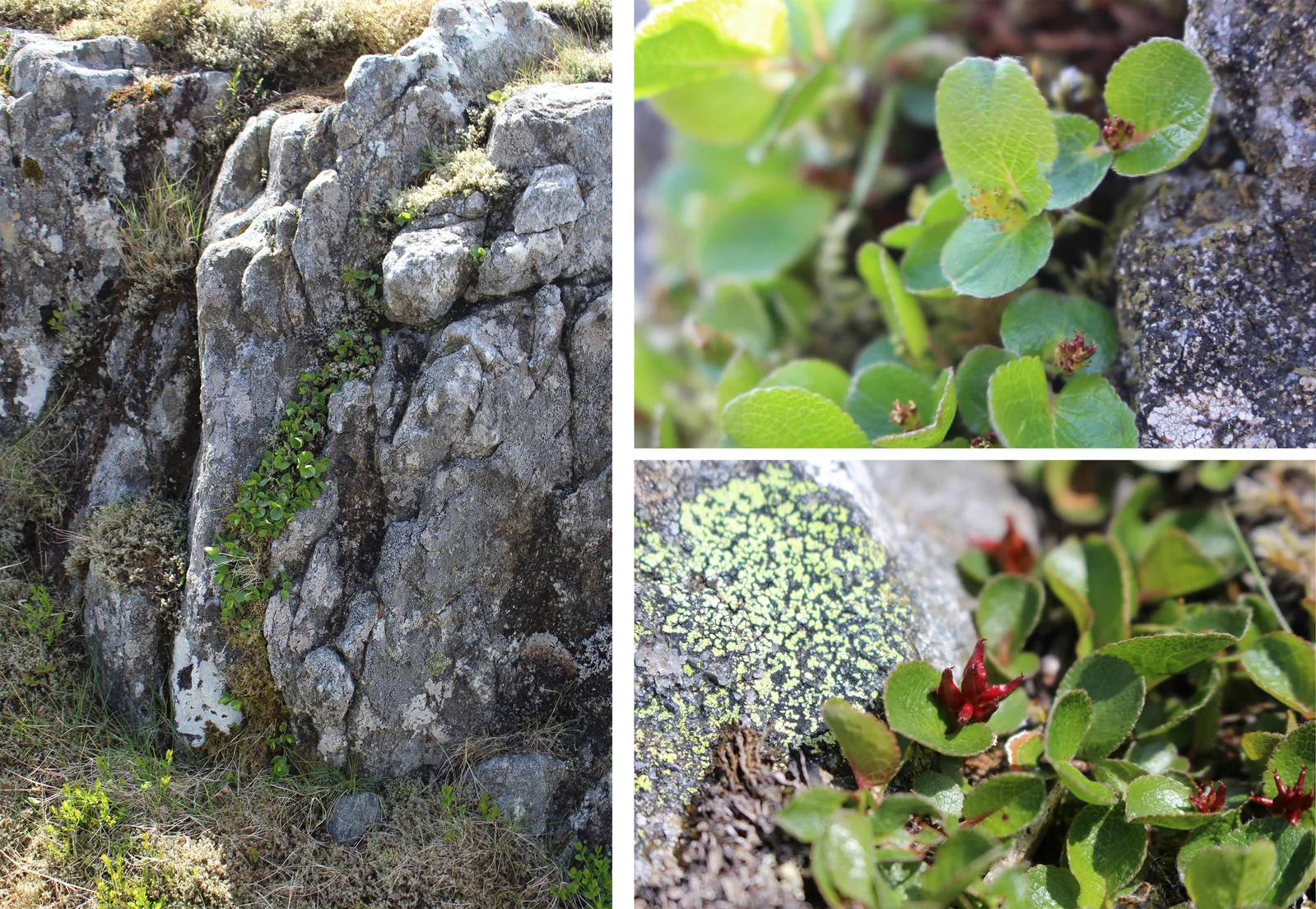
*Salix herbacea* (ddSalHerb6). Left, habitat; upper right, leaves and catkins; lower right, fruits. Photographs: Markus Ruhsam.

## Methods

### Sample acquisition, flow cytometry and DNA barcoding

A specimen of *S. herbacea* (specimen ID EDTOL01417, ToLID ddSalHerb6; Figure 1) was used for genome sequencing. It was collected from Fraoch-heinn, Scotland, UK (latitude 56.8911, longitude −5.467) on 2021-06-22. A second specimen was used for Hi-C sequencing (specimen ID EDTOL01412, ToLID ddSalHerb1). It was collected on the same occasion as the ddSalHerb6 specimen. The specimens were collected and identified by Markus Ruhsam. The ddSalHerb6 specimen was also used for RNA sequencing. The herbarium voucher for the sequenced plant (collector number DMR61) is deposited at Edinburgh ( E01152277).

The genome size was estimated by flow cytometry following the ‘one-step’ method outlined in Pellicer *et al*. (2021) and using propidium iodide as the fluorochrome. CyStain PI OxProtect Staining Buffer (Sysmex UK Ltd) was used for isolation of nuclei ( Loureiro *et al.*, 2007), and the internal calibration standard was *Solanum lycopersicum* L. ‘Stupické polní rané’ with an assumed 1C-value of 968 Mb ( Doležel *et al.*, 2007).

The initial identification was verified by an additional DNA barcoding process according to the framework developed by Twyford *et al*. (2024). Part of the plant specimen was preserved in silica gel desiccant ( Chase & Hills, 1991). DNA extracted from the dried plant was amplified by PCR for standard barcode markers, with the amplicons sequenced and compared to public sequence databases including GenBank and the Barcode of Life Database (BOLD) ( Ratnasingham & Hebert, 2007). Following whole-genome sequencing, the relevant DNA barcode region was also used alongside the initial barcoding data for sample tracking at the WSI ( Twyford *et al.* , 2024). The standard operating procedures for Darwin Tree of Life barcoding are available on protocols.io.

### Nucleic acid extraction

High molecular weight (HMW) DNA extraction protocols developed by the Tree of Life Core Laboratory at the Wellcome Sanger Institute (WSI) are available on protocols.io ( Howard *et al.*, 2025). Tissue from the ddSalHerb6 sample was weighed and triaged to select the appropriate extraction workflow.

HMW DNA was prepared for two PacBio HiFi libraries: DTOL13810174 and DTOL15031522. The tissue input, HMW DNA extraction, downstream fragmentation and clean-up, and post-shearing quality-control measurements for each PacBio library are summarised in Table 1.

The concentration of the sheared and purified DNA was assessed with a NanoDrop spectrophotometer and a Qubit Fluorometer using the Qubit dsDNA High Sensitivity Assay kit. Fragment size distribution was evaluated by running the sample on the Femto Pulse system.

**Table 1.** DNA extraction and post-extraction processing for each PacBio library used for *S. herbacea*.

**PacBio library ID**	**Tissue preparation**	**HMW DNA extraction**	**Fragmentation and clean-up**	**Post-shearing QC**
DTOL13810174	leaf; cryoPREP	Automated Plant Organic Extraction	Fragmentation: Megaruptor®3 for LI PacBio; clean-up: Automated SPRI	Qubit 14.3 ng/μL; DNA yield 1,647.00 ng; fragment size 13.7 kb
DTOL15031522	leaf; bead beating	Automated Plant Organic Extraction	Fragmentation: Megaruptor®3 for LI PacBio; clean-up: Automated SPRI	Qubit 12.8 ng/μL; DNA yield 1,404.00 ng; fragment size 19.4 kb

RNA was extracted from leaf tissue of ddSalHerb6 in the Tree of Life Laboratory at the WSI using the RNA Extraction: Automated MagMax™ *mir*Vana protocol. The RNA concentration was assessed with a NanoDrop spectrophotometer and a Qubit Fluorometer using the Qubit RNA Broad-Range Assay kit. RNA integrity was assessed using the Agilent RNA 6000 Pico Kit and Eukaryotic Total RNA assay.

### PacBio HiFi library preparation and sequencing

Library preparation and sequencing took place in the WSI Scientific Operations core. Libraries DTOL13810174 and DTOL15031522 were prepared with the SMRTbell Prep Kit 3.0 (Pacific Biosciences) according to the manufacturer’s instructions. The kit includes reagents for end repair/A-tailing, adapter ligation, post-ligation SMRTbell bead clean-up, and nuclease treatment. Size selection and clean-up were performed using diluted AMPure PB beads (Pacific Biosciences). DNA concentration was quantified with a Qubit Fluorometer v4.0 (Thermo Fisher Scientific) and the Qubit 1X dsDNA HS assay kit. Final library fragment size was assessed with the Agilent Femto Pulse Automated Pulsed Field CE Instrument (Agilent Technologies) using the gDNA 55 kb BAC analysis kit.

The libraries were sequenced on a Revio instrument (Pacific Biosciences, California, USA). The prepared libraries selected for multiplexing were pooled based on genome size, library molarity, and the intended plex level. Primers were annealed and polymerases were bound to generate circularised complexes, following the manufacturer’s instructions. Complexes were purified using SMRTbell beads, diluted to the Revio loading concentration, and spiked with a Revio sequencing internal control. The pooled libraries were sequenced on a Revio 25M Plex SMRT cell in a 4-plex run.

SMRT Link software (Pacific Biosciences), a web-based workflow manager, was used to configure and monitor the runs and to carry out primary and secondary data analysis.

### Hi-C

***Sample preparation and crosslinking***

Hi-C data were generated from the ddSalHerb1 leaf sample using the Arima-HiC v2 kit (Arima Genomics). Tissue was finely ground using the Covaris cryoPREP Dry Pulverizer (Covaris), and then subjected to nuclei isolation. Nuclei were isolated using a modified protocol based on the Qiagen QProteome Cell Compartment Kit (Qiagen), in which only the Lysis and CE2 buffers were used, with QIAshredder spin columns. After isolation, nuclei were fixed using formaldehyde to a final concentration of 2% to crosslink the DNA. The crosslinked DNA was then digested and biotinylated according to the manufacturer’s instructions. A clean-up step was performed with SPRIselect beads before library preparation. DNA concentration was quantified using the Qubit Fluorometer v4.0 (Thermo Fisher Scientific) and the Qubit HS Assay Kit, following the manufacturer’s instructions.

***Hi-C library preparation and sequencing***

Biotinylated DNA constructs were fragmented using a Covaris E220 sonicator and size-selected to 400–600 bp using SPRISelect beads. DNA was enriched with Arima-HiC v2 kit Enrichment beads. End repair, A-tailing, and adapter ligation were carried out with the NEBNext Ultra II DNA Library Prep Kit (New England Biolabs), following a modified protocol where library preparation occurs while DNA remains bound to the Enrichment beads. Libraries were amplified with KAPA HiFi HotStart mix and a custom Unique Dual Index (UDI) barcode set (Integrated DNA Technologies). Depending on sample concentration and biotinylation percentage determined at the crosslinking stage, libraries were amplified with 10–16 PCR cycles. Post-PCR clean-up was performed with SPRISelect beads. Libraries were quantified using the AccuClear Ultra High Sensitivity dsDNA Standards Assay Kit (Biotium) and a FLUOstar Omega plate reader (BMG Labtech).

Prior to sequencing, libraries were normalised to 10 ng/μL. Normalised libraries were quantified again to create equimolar and/or weighted 2.8 nM pools. Pool concentrations were checked using the Agilent 4200 TapeStation (Agilent) with High Sensitivity D500 reagents before sequencing. Libraries were sequenced on the Illumina NovaSeq 6000, generating paired-end 150-bp reads.

### RNA library preparation and sequencing

Libraries were prepared with the NEBNext® Ultra II Directional RNA Library Prep Kit for Illumina (New England Biolabs), following the manufacturer’s instructions. Poly(A) mRNA was isolated from 100 ng of total RNA using oligo(dT) beads, converted to cDNA, and uniquely indexed; 14 PCR cycles were performed. Libraries were prepared with an intended average fragment size of 100–300 bp. Libraries were quantified, normalised, and pooled equimolarly to a final concentration of 2.8 nM before dilution to 150 pM for loading. Sequencing was carried out on the Illumina NovaSeq 6000, generating paired-end reads.

### Genome assembly

Prior to assembly of the PacBio HiFi reads, a database of *k*-mer counts ( *k* = 31) was generated from the filtered reads using FastK. The *k*-mer frequency distribution was analysed with GenomeScope (version 2.1.0) ( Ranallo-Benavidez *et al.*, 2020), providing fitted point estimates of haploid genome size, heterozygosity and repeat content.

The HiFi reads were assembled using Hifiasm ( Cheng *et al.*, 2021) with the --primary option. The Hi-C reads ( Rao *et al.* , 2014) were mapped to contigs using bwa-mem2 ( Vasimuddin *et al.*, 2019), and the contigs were scaffolded in YaHS ( Zhou *et al.*, 2023) with the --break option for handling potential misassemblies. The scaffolded assemblies were evaluated using Gfastats ( Formenti *et al.* , 2022), BUSCO ( Tegenfeldt *et al.*, 2025) and MerquryFK ( Rhie *et al.*, 2020).

The organelle genomes were assembled using OATK ( Zhou *et al.*, 2025). The comparator for the mitochondrial assembly was the mitochondrial genome of *Salix pseudolasiogyne* (PQ873111.1). The comparator for the plastid assembly was the chloroplast genome of *Salix herbacea* (MW435436.1).

### Assembly curation

The assembly was decontaminated using the Assembly Screen for Cobionts and Contaminants ( ASCC) pipeline. The flat files and maps for curation were generated with TreeVal. Manual curation was conducted primarily in PretextView and HiGlass ( Kerpedjiev *et al.* , 2018). Scaffolds were visually inspected and corrected as described by Howe *et al*. (2021). The primary and alternate assemblies were combined for curation. Manual corrections included 24 breaks and 80 joins. This reduced the scaffold count by 21.5%, increased the scaffold N50 by 24.2%, and reduced the total assembly length by 4.8%. The curation process is described at sanger-tol/curation-resources. A Hi-C contact map of the primary assembly was generated with PretextSnapshot.

### Assembly quality assessment

The MerquryFK tool ( Rhie *et al.*, 2020) was run in a Singularity container ( Kurtzer *et al.*, 2017) to evaluate *k*-mer completeness and assembly quality for the primary and alternate haplotypes using the *k*-mer database ( *k* = 31) computed prior to genome assembly. The analysis outputs included assembly QV scores and completeness statistics.

The primary assembly was assessed using BlobToolKit ( Challis *et al.*, 2020). PacBio reads were mapped to the assembly to derive sequence coverage, and BUSCO ( Tegenfeldt *et al.*, 2025) was used to assess gene-set completeness. DIAMOND BLASTp searches of extracted BUSCO proteins and DIAMOND BLASTx searches of assembly sequences against UniProt Reference Proteomes ( Buchfink *et al.*, 2021), followed by BLASTn searches of sequences without DIAMOND BLASTx hits against the NCBI nt database ( Altschul *et al.*, 1990), supported taxonomic assignment. Coverage, sequence composition, taxonomic assignment and BUSCO results were integrated into a BlobDir for interactive inspection and visualisation. The snail and blob plots were rendered locally from this hosted BlobDir using BlobTk v0.8.3. The scaffold-length radial scale function for the snail plot was set to linear ( Challis & Blaxter, 2026). The blob plot used circular markers with area proportional to scaffold length ( Challis *et al.*, 2020).

## Genome sequence report

### Sequence data

The genome of a specimen of *S. herbacea* was sequenced using Pacific Biosciences single-molecule HiFi long reads, generating 22.29 Gb (gigabases) from 1.94 million reads. These reads were used to assemble the genome. GenomeScope analysis using a *p* = 2 model gave a *k*-mer-based haploid genome size estimate of 399.77 Mb, with heterozygosity of 1.64% and repeat content of 44.32%; the full-model fit was 97.15% (Figure 2). Using flow cytometry, the genome size (1C‐value) of the sample was estimated to be 0.51 pg, equivalent to 500.00 Mb. These estimates guided expectations for the assembly. The NCBI assembly record reports 53× genome coverage. Hi-C sequencing produced 90.18 Gb from 298.62 million read pairs, which were used to scaffold the primary assembly. RNA sequencing data were also generated and are available in public sequence repositories. Table 2 summarises the specimen and sequencing details.

**Figure 2.**
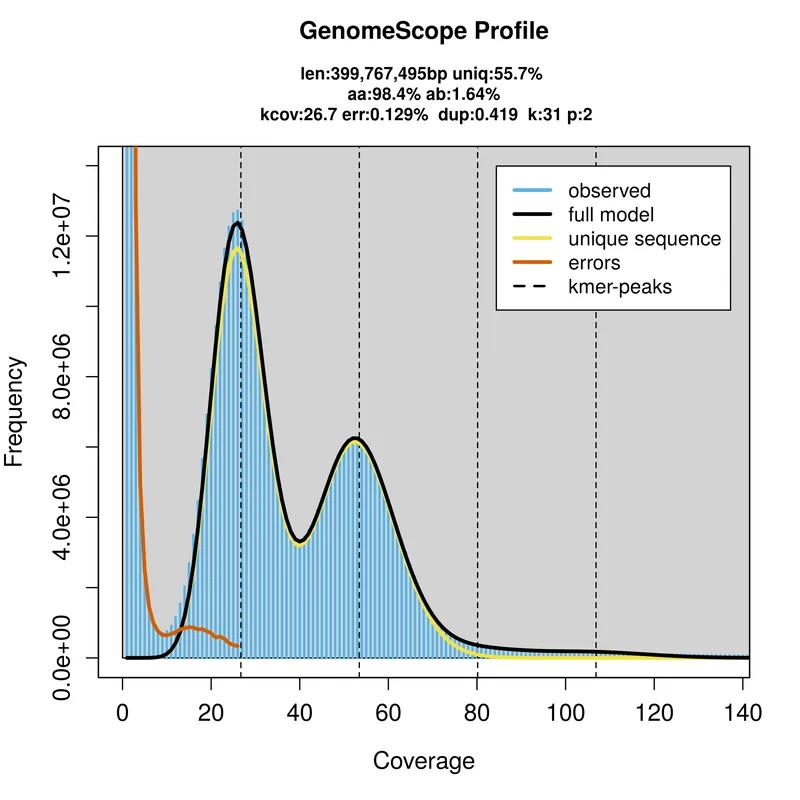
GenomeScope model fitted to the *k*-mer frequency distribution. The plot shows the observed and modelled *k*-mer spectra used to estimate haploid genome size, heterozygosity and repeat content from unassembled sequencing reads.

**Table 2.** Specimen and sequencing data for *S. herbacea* (BioProject PRJEB89302)

**Platform**	**PacBio HiFi**	**Hi-C**	**RNA-seq**
**ToLID**	ddSalHerb6	ddSalHerb1	ddSalHerb6
**Specimen ID**	EDTOL01417	EDTOL01412	EDTOL01417
**BioSample (source individual)**	SAMEA9335576	SAMEA9335571	SAMEA9335576
**BioSample (tissue)**	SAMEA9335599	SAMEA9335581	SAMEA9335599
**Tissue**	leaf	leaf	leaf
**Instrument**	Revio (2 runs)	Illumina NovaSeq 6000	Illumina NovaSeq 6000
**Library ID & run accession(s)**	DTOL13810174: ERR14955664; DTOL15031522: ERR14955666	DN847693C:H1: ERR14986738	ERR14986739
**Read count total**	1.94 million reads	298.62 million read pairs	24.66 million read pairs
**Base count total**	22.29 Gb	90.18 Gb	7.45 Gb

### Assembly statistics

The primary assembly was generated and an alternate haplotype assembly was also deposited at scaffold level in INSDC databases. The primary assembly is proposed as the reference assembly for this species. It has a total length of 421.43 Mb in 155 scaffolds (excluding organelle sequences), with 51 gaps, and its scaffold N50 is 21.4 Mb (Table 3). The scaffold auN for the primary assembly is 20.2 Mb.

**Table 3.** Genome assembly statistics for *S. herbacea*

**Genome assembly**	**Primary assembly**	**Alternate haplotype assembly**
**Assembly name**	ddSalHerb6.1	ddSalHerb6.1 alternate haplotype
**Assembly accession**	GCA_965644465.1	GCA_965644525.1
**Assembly level**	chromosome	scaffold
**Span (Mb)**	421.43	387.36
**Number of chromosomes**	19	-
**Number of contigs**	206	981
**Contig N50 (Mb)**	9.63	2.55
**Number of scaffolds (excluding organelle sequences)**	155	706
**Scaffold N50 (Mb)**	21.4	18.15
**Scaffold auN (Mb)**	20.2	-
**Longest scaffold length (Mb)**	32.81	32.07
**Organelles**	Mitochondrial genome: 692.62 kb; Plastid genome: 155.63 kb	-

Most of the primary assembly sequence (92.79%) was assigned to 19 chromosome-level scaffolds. These chromosome-level scaffolds, confirmed by Hi-C data, are named according to size (Figure 3; Table 4).

The mitochondrial genome (length 692.62 kb, OZ286670.1) and plastid genome (length 155.63 kb, OZ286671.1) were also assembled. These sequences are included as contigs in the multifasta file of the primary genome submission and as standalone records.

**Figure 3.**
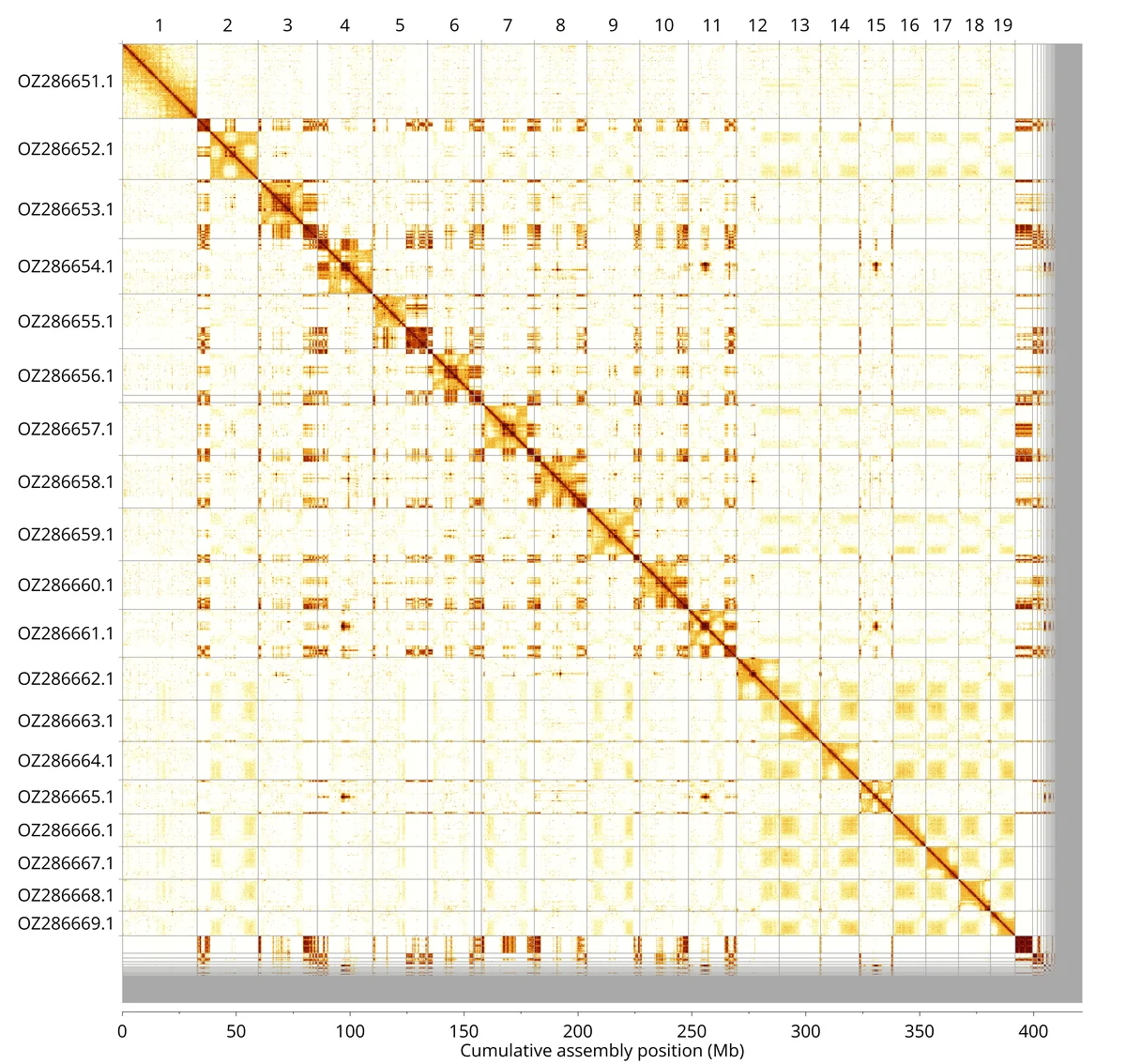
Hi-C contact map of the *S. herbacea* primary assembly. Assembled chromosomes are shown in order of size and labelled along the axes, with a megabase scale shown below. The plot was generated using PretextSnapshot.

**Table 4.** Chromosomal pseudomolecules in the primary genome assembly of *S. herbacea* ddSalHerb6

**INSDC accession**	**Molecule**	**Length (Mb)**	**GC%**
OZ286651.1	1	32.81	33.50
OZ286652.1	2	26.75	35
OZ286653.1	3	25.93	35.50
OZ286654.1	4	24.16	37
OZ286655.1	5	24.06	35.50
OZ286656.1	6	23.65	34.50
OZ286657.1	7	23.14	35
OZ286658.1	8	23.06	35
OZ286659.1	9	23.06	34.50
OZ286660.1	10	21.40	35
OZ286661.1	11	20.95	38
OZ286662.1	12	18.70	34.50
OZ286663.1	13	18.13	34.50
OZ286664.1	14	16.87	35
OZ286665.1	15	14.91	37
OZ286666.1	16	14.34	35
OZ286667.1	17	14.31	34
OZ286668.1	18	14.02	34.50
OZ286669.1	19	10.79	34.50

### Assembly quality metrics

The combined primary and alternate assemblies achieve an estimated QV of 62.8. The *k*-mer completeness is 71.51% for the primary assembly, 70.87% for the alternate haplotype, and 99.05% for the combined assemblies (Figure 4).

BUSCO v.6.0.0 analysis of the primary assembly using the eudicots_odb10 reference set ( *n* = 2,326) identified 98.9% of the expected gene set (single = 84.3%, duplicated = 14.6%).

**Figure 4.**
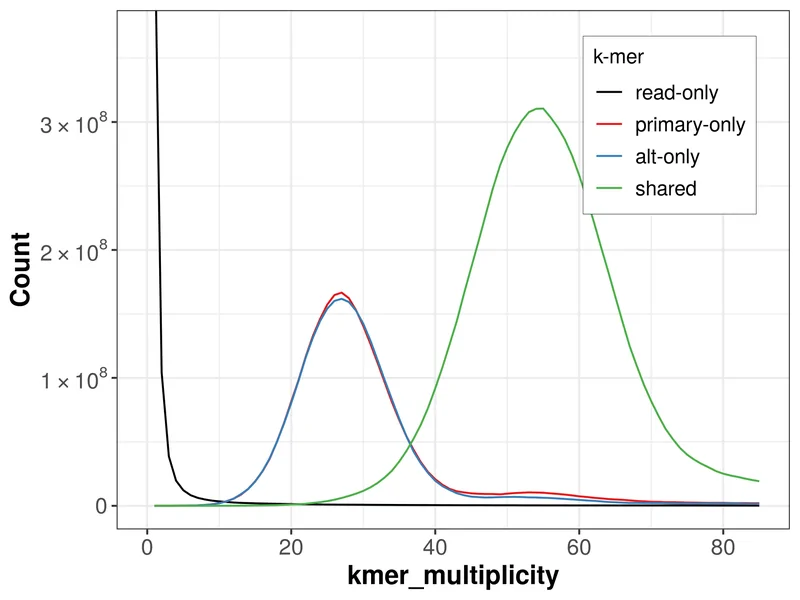
Evaluation of *k*-mer completeness using MerquryFK. This plot illustrates the recovery of *k*‐mers from the original read data in the primary assembly, the alternate haplotype assembly and the combined assemblies. The horizontal axis represents *k*‐mer multiplicity, and the vertical axis shows the number of *k*‐mers. The black curve represents *k*‐mers that appear in the reads but are not assembled, while the remaining curves correspond to *k*‐mers recovered in the assemblies shown.

The snail plot in Figure 5 provides a summary of assembly contiguity, cumulative scaffold count, sequence composition and BUSCO completeness for the primary assembly ( Challis & Blaxter, 2026). The relative sizes of the longest scaffold, scaffold N50 and N90, together with the dark-grey scaffold-length distribution, show how much of the assembly is contained in long scaffolds. The outer composition track shows variation in GC, AT and N content, while the central track shows the cumulative scaffold count and the BUSCO inset summarises gene-set completeness.

The blob plot in Figure 6 displays scaffolds from the primary assembly by GC proportion and base coverage. Marker area is proportional to scaffold length, and colour indicates taxonomic assignment ( Challis *et al.*, 2020).

**Figure 5.**
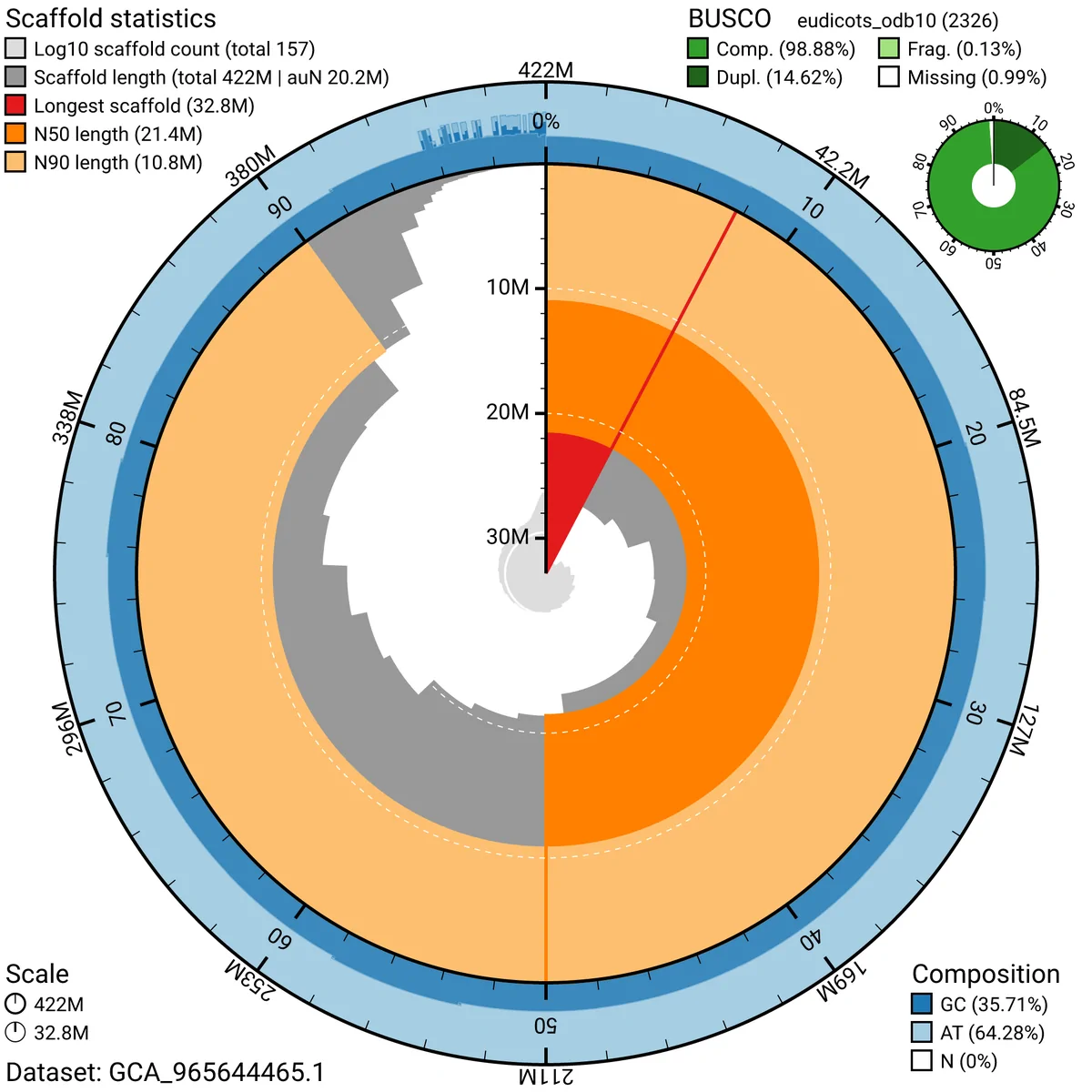
Assembly metrics for ddSalHerb6.1, the primary assembly. The BlobToolKit snail plot provides an overview of assembly metrics and BUSCO gene completeness. The circumference represents the length of the primary assembly, and the main plot is divided into 1,000 bins around the circumference. The outermost blue tracks display the distribution of GC, AT, and N percentages across the bins. Scaffolds are arranged clockwise from longest to shortest and are depicted in dark grey. Scaffold length is shown on a linear radial scale, scaled to the longest scaffold. Red shading indicates the longest scaffold, and the deeper orange and pale orange shading represent the N50 and N90 lengths. The summary statistics include scaffold auN, the area under the scaffold Nx curve. The light grey spiral at the centre shows the cumulative scaffold count on a logarithmic scale. A summary of complete, fragmented, duplicated, and missing BUSCO genes in the eudicots_odb10 set is presented at the top right. The assembly can be explored interactively in the BlobToolKit viewer.

**Figure 6.**
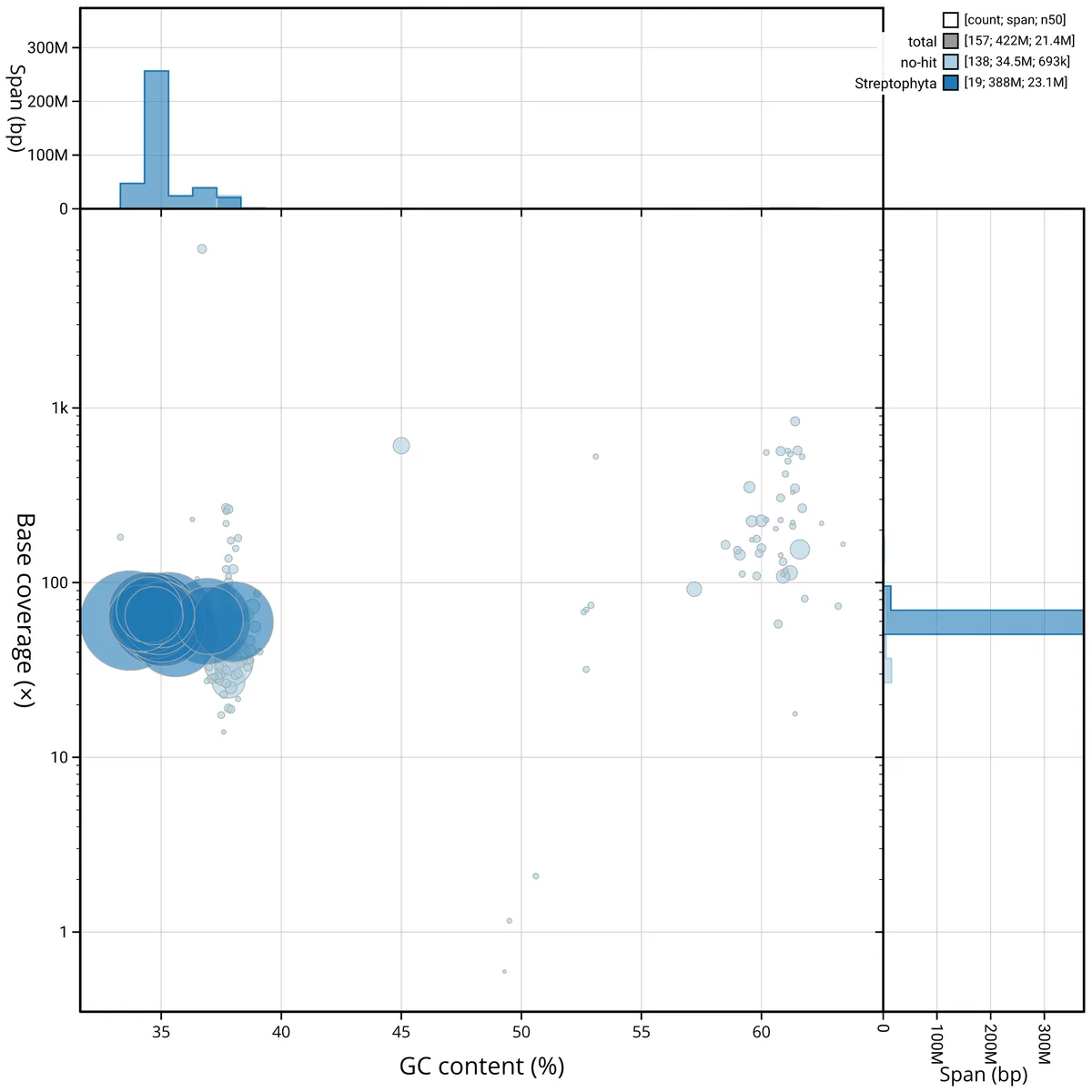
BlobToolKit blob plot for ddSalHerb6.1. The plot shows base coverage (vertical axis) and GC content (horizontal axis). Circles represent scaffolds; marker area is proportional to scaffold length, and colour indicates taxonomic assignment. The histograms along the axes display the total length of sequences distributed across different levels of coverage and GC content. The assembly can be explored interactively in the BlobToolKit viewer.

Table 5 lists the assembly metric benchmarks adapted from Rhie *et al.*. (2021) and the Earth BioGenome Project (EBP) Report on Assembly Standards. The three-part EBP metric calculated for the primary assembly is **6.C.Q63**.

**Table 5.** Earth BioGenome Project summary metrics for the *S. herbacea* assembly

**Measure**	**Value**	***Benchmark***
EBP summary (primary)	6.C.Q63	*6.C.Q40*
Contig N50 length	9.63 Mb	*> 1 Mb*
Scaffold N50 length	21.40 Mb	*= chromosome N50*
Consensus quality (QV)	Primary: 63.6; alternate: 62.4; combined: 62.8	*≥ 40*
*k*-mer completeness	Primary: 71.51%; alternate: 70.87%; combined: 99.05%	*> 90% (combined assembly)*
BUSCO	C:98.9% [S:84.3%, D:14.6%], F:0.1%, M:1.0%, n:2,326	*C > 90%*
Percentage of assembly assigned to chromosomes	92.79%	*≥ 90%*

**Notes:** The EBP summary uses log10(Contig N50); chromosome-level (C) or log10(Scaffold N50); Q (Merqury QV). For *k*-mer completeness, the benchmark is assessed using the combined assembly value. BUSCO v6.0.0 using the eudicots_odb10 lineage dataset: C=complete; S=single-copy; D=duplicated; F=fragmented; M=missing; n=orthologues.

### Data availability

European Nucleotide Archive: Salix herbacea, ddSalHerb. Accession number PRJEB89302; https://identifiers.org/ena.embl/PRJEB89302. The genome sequence is released openly for reuse. The *S. herbacea* genome sequencing initiative is part of the Darwin Tree of Life Project ( PRJEB40665), the Sanger Institute Tree of Life Programme ( PRJEB43745) and AEGIS ( PRJEB80366). All raw sequence data and the primary and alternate assemblies have been deposited in INSDC databases. The genome will be annotated using available RNA-Seq data and presented through the Ensembl pipeline at the European Bioinformatics Institute. Raw data and assembly accession identifiers are reported in Table 2 and Table 3.

Pipelines used for genome assembly at the WSI Tree of Life are available at https://pipelines.tol.sanger.ac.uk/pipelines. Table 6 lists software versions used in this study.

**Table 6.** Software versions and sources

**Software**	**Version**	**Source**
BLAST	2.14.0	https://ftp.ncbi.nlm.nih.gov/blast/executables/blast+/
BlobToolKit	4.4.6	https://github.com/genomehubs/blobtoolkit
BlobTk (hosted BlobDir)	0.5.1	https://github.com/genomehubs/blobtk
BlobTk (snail and blob plot renderer)	0.8.3	https://github.com/genomehubs/blobtk
BUSCO	6.0.0; 6.1.0	https://gitlab.com/ezlab/busco
bwa-mem2	2.2.1	https://github.com/bwa-mem2/bwa-mem2
DIAMOND	2.1.8	https://github.com/bbuchfink/diamond
fasta_windows	0.2.4	https://github.com/tolkit/fasta_windows
FastK	1.2	https://github.com/thegenemyers/FASTK
GenomeScope	2.1.0	https://github.com/tbenavi1/genomescope2.0
Gfastats	1.3.6	https://github.com/vgl-hub/gfastats
Hifiasm	0.19.8-r603	https://github.com/chhylp123/hifiasm
HiGlass	1.13.4	https://github.com/higlass/higlass
MerquryFK	1.1.0-c1	https://github.com/thegenemyers/MERQURY.FK
Minimap2	2.28-r1209	https://github.com/lh3/minimap2
Oatk	0.9	https://github.com/c-zhou/oatk
MultiQC	1.14; 1.17 and 1.18	https://github.com/MultiQC/MultiQC
Nextflow	24.10.4	https://github.com/nextflow-io/nextflow
PretextSnapshot	0.0.6	https://github.com/sanger-tol/PretextSnapshot
PretextView	1.0.3	https://github.com/sanger-tol/PretextView
samtools	1.21	https://github.com/samtools/samtools
sanger-tol/ascc	0.1.0	https://github.com/sanger-tol/ascc
sanger-tol/blobtoolkit	v0.9.0	https://github.com/sanger-tol/blobtoolkit
sanger-tol/curationpretext	1.4.2	https://github.com/sanger-tol/curationpretext
Seqtk	1.4-r122	https://github.com/lh3/seqtk
Singularity	3.9.0	https://github.com/sylabs/singularity
TreeVal	1.3.0	https://github.com/sanger-tol/treeval
YaHS	1.2.2	https://github.com/c-zhou/yahs

### Author information

- Members of the Royal Botanic Garden Edinburgh Genome Acquisition Lab
- Members of the Plant Genome Sizing Collective
- Members of the Darwin Tree of Life Barcoding collective
- Members of the Wellcome Sanger Institute Tree of Life Management, Samples and Laboratory team
- Members of Wellcome Sanger Institute Scientific Operations – Sequencing Operations
- Members of the Wellcome Sanger Institute Tree of Life Core Informatics team
- Members of the Tree of Life Core Informatics collective
- Members of the Darwin Tree of Life Consortium

### Wellcome Sanger Institute – Legal and Governance

The materials that have contributed to this genome note have been supplied by a Darwin Tree of Life Partner. The submission of materials by a Darwin Tree of Life Partner is subject to the **‘Darwin Tree of Life Project Sampling Code of Practice’**, which can be found in full on the Darwin Tree of Life website. By agreeing with and signing up to the Sampling Code of Practice, the Darwin Tree of Life Partner agrees they will meet the legal and ethical requirements and standards set out within this document in respect of all samples acquired for, and supplied to, the Darwin Tree of Life Project. Further, the Wellcome Sanger Institute employs a process whereby due diligence is carried out proportionate to the nature of the materials themselves, and the circumstances under which they have been/are to be collected and provided for use. The purpose of this is to address and mitigate any potential legal and/or ethical implications of receipt and use of the materials as part of the research project, and to ensure that in doing so we align with best practice wherever possible. The overarching areas of consideration are:

- Ethical review of provenance and sourcing of the material
- Legality of collection, transfer and use (national and international)

Each transfer of samples is further undertaken according to a Research Collaboration Agreement or Material Transfer Agreement entered into by the Darwin Tree of Life Partner, Genome Research Limited (operating as the Wellcome Sanger Institute), and in some circumstances, other Darwin Tree of Life collaborators.
