# Supplementary material for: The genome sequence of the Dwarf Willow, Salix herbacea L. (Malpighiales: Salicaceae)
Source: Wellcome Open Res. 2026 Oct 5;11:808. [Version 1] doi: 10.12688/wellcomeopenres.28051.1 (PMC13675631; doi:10.12688/wellcomeopenres.28051.1)
Supplement: Peer review history

# Reviewer report: Reviewer response for version 1

**DOI:** 10.21956/wellcomeopenres.30857.r172541

**Published:** 2026-10-16

**Authors:** Xue-Jun Ge

**Affiliations:** 1 Chinese Academy of Sciences, Guangzhou, China

This study presents a chromosome-level genome assembly of *Salix herbacea* , supported by PacBio HiFi sequencing and Hi-C scaffolding. The assembly shows high completeness and represents a potentially valuable genomic resource. However, several aspects of the assembly workflow require further clarification.

**Major comments**

**1. Please provide more details on the generation of the alternate haplotype assembly.**

 The manuscript primarily describes the workflow used to generate the primary assembly, while the alternate haplotype assembly is mentioned several times and appears to have been involved in manual curation. The authors should clarify how the alternate haplotype assembly was generated, including the software, key parameters, and subsequent processing steps.

**2** **. Please clarify the k-mer completeness of the primary assembly and report its Merqury QV separately.**

 The k-mer completeness is 71.51% for the primary assembly, compared with 99.05% for the combined primary and alternate assemblies. This difference may partly reflect sequence differences between the two haplotypes; however, these metrics alone do not establish whether the primary assembly contains substantial sequence omissions. The authors should clarify the likely explanation for this result and discuss its implications for the use of the primary assembly as a reference genome.

 In addition, the reported Merqury QV appears to refer to the combined assemblies. If possible, the authors should report the QV of the primary assembly separately to provide a more direct assessment of the base-level accuracy of the proposed reference genome.

**3** **. Please provide additional evidence to validate the accuracy of Hi-C scaffolding.**

 The Hi-C contact map in Figure 3 shows relatively clear diagonal patterns across the 19 chromosomes, but also contains numerous off-diagonal interaction signals and localized high-intensity blocks, particularly in the regions involving Chr2–Chr11 and in Chr15. These patterns may reflect repetitive sequences, ambiguous read mapping, or potential assembly errors.

 The authors should clarify whether these signals were investigated during manual curation and provide additional evidence to support the accuracy of chromosome assignments and scaffold orientations. Synteny analysis with closely related species would be helpful for evaluating chromosome-level organization and identifying potential structural inconsistencies. If further analyses indicate potential scaffolding errors, the authors could consider reassessing the relevant regions using Hi-C scaffolding tools such as 3D-DNA, or Haphic, and describe the rationale for the final corrections.

**4** **. Please report the read mapping rate to further support the assembly quality assessment.**

 The authors report several assembly quality metrics, including k-mer completeness, Merqury QV, and BUSCO completeness. As an additional assessment, I recommend mapping the original PacBio HiFi reads to the final primary assembly using Minimap2/BWA and reporting the read mapping rate. This would provide complementary evidence regarding how well the assembly represents the sequencing data.

**Minor comment**

**1** **. Please define auN and clarify how it is calculated.**

 The authors should provide the full name of auN in the genome assembly statistics.

**2** **. Figure 5**

 To improve readability, the chromosome should be labeled on the outermost ring. In addition, the BUSCO results should be presented as four categories: Single, Duplicated, Fragmented, and Missing, to provide a more comprehensive and intuitive summary of genome assembly completeness.

**3** **. Figure 6**

 The legend in the upper-right corner overlaps with the plotted content and should be repositioned or the layout adjusted to avoid obscuring the figure. In addition, the y-axis labels should be rotated by 180° to match the orientation used in the preceding figures, thereby improving consistency and readability.

Are the datasets clearly presented in a usable and accessible format, and the assembly and annotation available in an appropriate subject-specific repository?

Yes

Are sufficient details of the sequencing and extraction, software used, and materials provided to allow replication by others?

Yes

Are the rationale for sequencing the genome and the species significance clearly described?

Yes

Are the protocols appropriate and is the work technically sound?

Yes

Reviewer Expertise:

Plant Phylogeny, and conservation genomics

I confirm that I have read this submission and believe that I have an appropriate level of expertise to confirm that it is of an acceptable scientific standard.

# Reviewer report: Reviewer response for version 1

**Published:** 2026-10-05

**Authors:** Automated Benchmarking

**Affiliations:** 1 Wellcome Sanger Institute, Hinxton, UK

As this article is a Genome Note, Automated Benchmarking has been used to determine whether the reported assembly passes four defined checks established by the Earth BioGenome Project: the raw and assembled data are publicly available, the Earth BioGenome Project three-part metric is met, the k-mer completeness exceeds 90%, and the BUSCO score passes the stated threshold. For Salix herbacea (ddSalHerb6), all four checks passed. Data available: pass. ENA study PRJEB89302, raw-read accessions ERR14955664, ERR14955666, ERR14986738, and ERR14986739, and assembly accessions GCA_965644465.1 and GCA_965644525.1 are public. Earth BioGenome Project three-part metric: pass. The primary assembly is reported as 6.C.Q63, meeting the 6.C.Q40 reference standard. k-mer completeness: pass. The combined haplotype assemblies have 99.05% k-mer completeness, exceeding the Earth BioGenome Project target of 90%. BUSCO: pass. The primary assembly, which is proposed as the reference assembly for this species, has 98.9% complete BUSCOs (84.3% single-copy and 14.6% duplicated). BUSCO software version: 6.0.0; lineage dataset: eudicots_odb10. It meets the plant-specific target of greater than 90% complete BUSCOs. This decision uses values reported in the Genome Note and public accession records.

Reviewer Expertise:

NA

I confirm that I have read this submission and believe that I have an appropriate level of expertise to confirm that it is of an acceptable scientific standard.
